# Software development skills for health data researchers

**DOI:** 10.1136/bmjhci-2021-100488

**Published:** 2022-08-09

**Authors:** Caroline Morton, Nicholas Devito, Jessica Morley, Iain Dillingham, Anna Schultze, Sebastian Bacon, Peter Inglesby, Steven Maude, Ben Goldacre

**Affiliations:** 1 Nuffield Department of Primary Care Health Sciences, University of Oxford, Oxford, Oxfordshire, UK; 2 University of Oxford, Oxford, Oxfordshire, UK; 3 Faculty of Epidemiology and Population Health, London School of Hygiene & Tropical Medicine, London, UK; 4 Primary Care Health Sciences, University of Oxford, Oxford, Oxfordshire, UK

**Keywords:** programming languages, medical informatics computing, data management, data science, documentation

## Introduction

Health data researchers are increasingly required to develop complex analytic code in order to implement sophisticated analyses on large health datasets. While writing analysis scripts (box 1) for academic projects is distinct from general purpose software development, they share many of the same features. A researcher’s script usually consists of a sequence of commands executed by a computer to extract, reshape, clean, describe and analyse data. If the quality of this analytic code cannot be reasonably assured, then results cannot be trusted: programming errors have resulted in high profile retractions.^1–3^ Similarly, if lengthy scripts for data management cannot be re-used, then work is needlessly duplicated.

Box 1 **Glossary**

**Analytic Script:** A series of commands written in a programming or statistical language such as R, Stata or Python, that are executed by a computer. These commands are used to *do* the analysis and may involve data extraction, cleaning, processing and analysis.

**Commit:** An individual change or revision to a file or set of files^9^

**Docstring:** This is a non-executable text that is attached to units of code such as functions, and documents what the code is doing. For example, this may include inputs, outputs, and specific errors.

**Functions:** These are pieces of code that can be run (or invoked) and executes the code specified.

**Library:** This is a collection of code that does a particular task or set of tasks, and can be imported and used in other projects.

**Open source:** Code or software projects where the source code is freely available and may be changed, and shared by others.

**Pull:** This is the term that describes when you fetch files from GitHub or similar. You can “pull” the most up to date file onto your computer, or “pull” changes that your colleague may have made.^9^

**Pull Request:** There are proposed changes to a repository by a user and are accepted or rejected, or commented on by the other project collaborators.^9^

**Push:** This is the term that describes when you send your committed changes back to GitHub (or a similar platform). Once pushed, others will be able to see your suggested changes to any files.^9^

**Repository:** This is a project space within GitHub or GitLab that holds a project. The easiest way of conceptualising this is as a folder that contains all your project files, and stores each files’ revision history.^9^

**Requirements/Dependencies**: These are software libraries that are required to run a particular project or piece of code. They normally have a version number, for example, version 0.0.1, 0.0.2 etc

The software engineering community has developed a range of techniques to improve the quality, re-usability, efficiency and readability of code. Organisations such as the Software Sustainability Institute^4^ support this approach to code development and provide more detailed guidance and education which are well worth reviewing. In this brief guide we explain how researchers can borrow best practices and freely available tools from this community to improve their work. We specifically cover the following three topics: Writing High Quality Code, Working Collaboratively and Sharing your work. Throughout the piece we often refer to examples from Python or R, two popular open source programming languages used by academics, but our advice is universal and there will be analogues to these examples in any commonly used statistical or general purpose programming language.

## Methods

In this section, we introduce the three major themes and break down each theme with some key concepts and practical guidance.

### Writing high quality code

Writing high quality code goes beyond the complexities of the analytic script itself, and should include documentation on what the code does, what decisions were taken and where, and how to recreate the same scripting environment in which the code runs. It can also include introducing efficiencies by encapsulating repeated code into functions that can be reused by you and others. Many programming languages also have style standards and specific recommendations on how to format and construct your code, like PEP8 for Python^5^ and the tidyverse for R^6^. While the specifics of these for any individual language are outside the scope of this article, it is worth looking into to make sure your code is readable and quickly understandable to others. Integrated Development Environments (IDEs) such as PyCharm and R Studio are software applications that can integrate the coding standards and highlight places in your code where these standards are violated. They also provide a number of other useful features that can help you work more effectively and efficiently such as syntax highlighting, code autocompletion, code search, and tools to find errors and run unit tests.

#### Documentation

Analytic scripts can be long and complex, and good documentation can improve reusability and understanding by providing information about what each section of the scripts is doing, and why. Increasing the readability of the code improves your user’s understanding, increases the likelihood that other people will use your code, and acts as an aide memoire when you return to your work after a period of time.

##### How to write and share good documentation

The simplest form of documentation is as a “comment” in-line with the code: these are text notes embedded in the code, marked so as not to be executed, that provide plain-English context for what is occurring in the adjacent commands. If your code is complex, and you have converted repeating code patterns into “functions” (as discussed below), then you can also build more formal documentation attached to these functions; in Python, for example, these are called “docstrings”. These are less like incidental comments for a few lines of code, and more like formal documentation that describes how a particular block of code can be invoked and used. Ideally, all code would also have some overarching contextual documentation. For researchers we recommend that this should include at minimum: simple instructions, including the order in which programmes should be run; project details (called a “readme” file); and a link to the research protocol. Ideally it should have enough information for a researcher to be able to “recreate” the software environment in which the research was run in (see below for more information on environments).

#### Cataloguing your environment

Analysis scripts and other forms of software do not exist in isolation: they are written to be executed in particular environments. A snapshot file, such as Python’s “requirements.txt”, captures those assumptions, to tell users the exact version of the programming language or statistical analysis packages (often called dependencies) that are needed for the code to execute. When executing code in a “walled garden” environment (such as the Stata software with no bespoke added libraries) it is sufficient to simply give the version number of the single piece of software used; in more complex environments, good cataloguing is vital. Software is constantly evolving and advancing; commands that once worked in a certain way may have changed their implementation, such that there are small or large differences in the output from a given command. By providing adequate information about the requirements of your code, someone else can accurately run your code.

##### How to Catalogue your environment

The exact name and process for creating a requirements file can vary by programming language but the idea is the same. Sometimes this documentation takes the form of a simple text file in your project repository that lists the software packages used, and their version numbers. This can be generated manually, but for complex environments and repeated use it is often better to automate cataloguing with tools such as “pip-tools” for Python. Other tools exist for more advanced users to create reproducible virtual environments or full virtual machines like Docker.^7 8^

#### Functions

It is common for the same task to be performed many times over in a given analysis, or across projects. Inexperienced coders will often copy and paste code “patterns”, with minor changes, to perform repetitive tasks. More experienced programmers aim to replace these code patterns with reusable “functions”, which group the repetitive tasks together into single units of code with their associated documentation. Using functions has the obvious benefit of reducing the risk of errors when having to make small changes to a part of the code, as the changes are made once within a function.

##### How to write a function

We use the term “function” here for simplicity: however the exact names and mechanisms for creating this kind of reusable code will vary by language and purpose (for example, “macros” in Stata are essentially the same as functions). All methods tend to share the same basic structure: creating generalisable code that takes defined inputs, executes, and then returns a standard output.

#### Unit tests

When repetitive tasks are grouped together into functions, these functions can be more easily “tested” to check that their observed behaviour matches their expected behaviour. Performing these checks manually is tedious and error-prone for humans, so programming languages provide additional tools to automate this process. Central to these tools are “unit tests”: pieces of code that systematically test a “unit” of code such as a function. They provide the function to be tested with a range of controlled inputs and allow the programmer to make assertions about the expected outputs, to verify that the function is performing as expected.

##### How to write a unit test

Tests are important: they allow you to change small parts of a complex analytic codebase confidently, with a safety net, knowing that many errors will be caught early. The programmer can run tests individually or in groups when writing code. There are also automatic integrations via platforms like GitHub or GitLab that run tests automatically each time new code is committed. It is a good idea to follow the “Arrange, Act, Assert” principle.^8^ *Arrange* a suitable curated input for the function to be tested on: for example, if the function transforms data, then recreate a much smaller version of that dataset where the correct function output has been pre-calculated. Then *Act* by passing this pre-prepared test dataset to the function that is being tested, and record the answer. Lastly, *Assert*: compare the output you got from the tested function with your earlier calculation. This could be done manually or preferably via code to assert that these two outputs match each other.

### Working collaboratively

Software engineers and health data researchers usually work in teams and need to collaborate effectively. Software engineers are well-versed in using tools such as GitHub for collaborative working, and these tools have a low barrier to entry for health-data researchers. In this section, we will introduce GitHub and how it can facilitate best practices of version control, and code review, within a team.

#### Using Github to share and manage code

All of the working practices described in this paper are supported by commonly used software tools, of which GitHub is the most prevalent. The key to good practice in software development is the use of a strong platform that facilitates iterative development with version control, code review, unit testing, and code sharing.^9^ GitHub is a good option as it is freely available for both private and public projects, well documented and supported, and friendly to beginners; other good alternatives such as GitLab also exist.

##### How to get started with GitHub

Users can sign up via www.github.com and make free accounts. This gives unlimited space for projects called repositories. Research groups may benefit from more advanced functionality that do have some associated costs. Projects can be changed from private to public, and vice versa, so it is possible to develop your code in private, and then share on publication of the associated paper, if that is a preferred pipeline.

#### Version control

Version control is the process of tracking and managing a project’s code throughout its development. Software platforms keep track of all changes made to the code and allow multiple researchers to work on the same code at the same time. Changes can then be merged back into one “main” codebase. Archives of these changes are automatically logged for future reference, with a record of who made each change; and changes to sections of code can be visualised for ease of comparison. It also provides a safety net, as code can easily be reverted back to an earlier version if a problem is encountered later on in the project.

##### How to do version control

**Figure 1:**
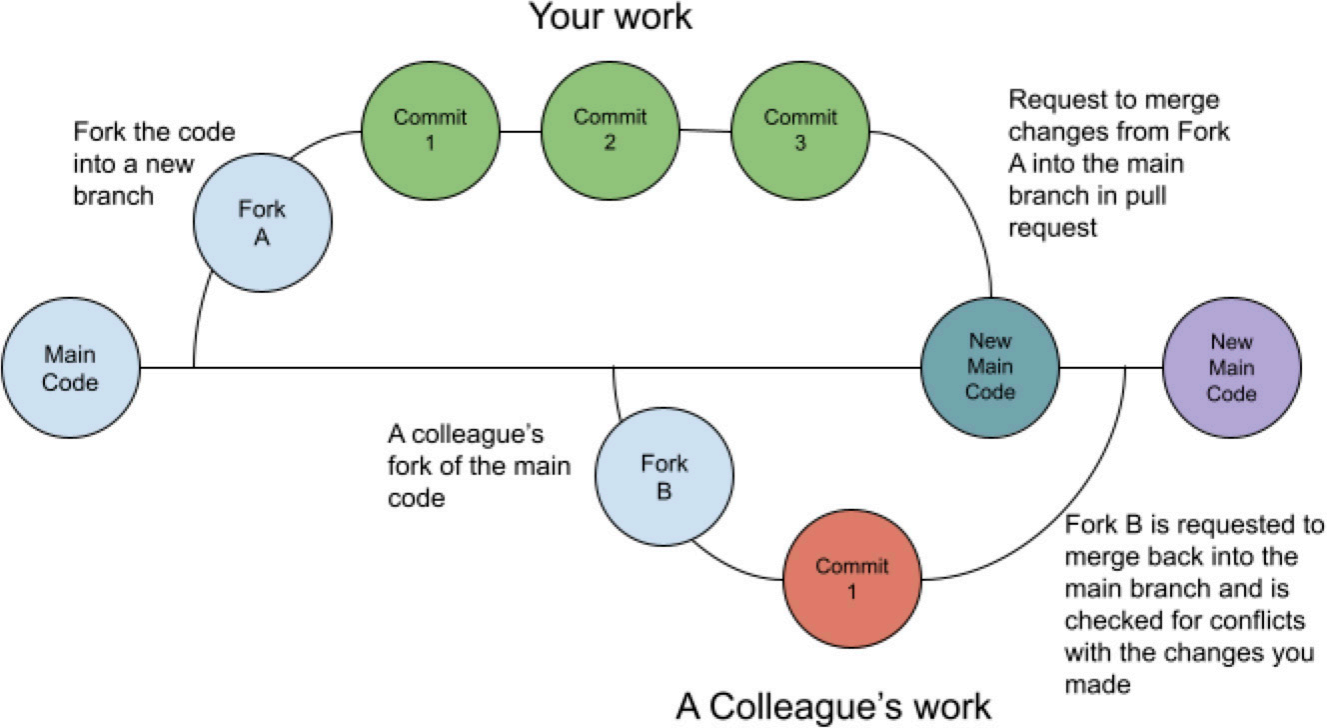
This figure shows an example workflow for a colleague and you using git to work on the same repository. In it, you fork your code (copy the repo) to work safely on the code whilst the current main branch remains untouched. You commit your changes and request to merge them back into the main branch. If accepted, these changes become part of the main code. Future merges by colleagues will be checked for conflicts since they were working on an earlier version of the code.

GitHub and other similar platforms facilitate version control as a built-in feature. Small changes to the code are submitted (called “commits”) and tracked. During development you can “clone” a copy of the repository to safely work on while the current codebase remains untouched. While users are pointed to the stable main code “branch”, you can safely revise, update, and experiment with your code until you are ready to commit the changes (figure 1).

**Figure 2:**
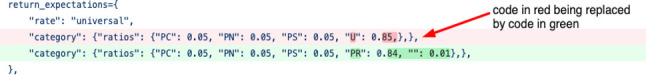
This screenshot of a pull request compares new code against existing code in the browser on GitHub. It shows proposed “new” code additions or edits in green, and code that is being removed or changed in red. Code that has not changed remains white.

Often you will propose changes to a repository in a “pull request” that documents all the edits you have made and are now proposing to be written over the canonical “main” version of the code. These pull requests act as a natural inflection point to ask for a code review (see below), and ensure none of your changes conflict with the current state of the repository. When a pull request is accepted and “merged” a history of all commits are maintained within the repository. This allows users to revisit any prior development state of the code, and provides transparency into the development of the project (figure 2).

#### Code review

Code will often contain shortcomings, or errors. A single incorrect character may have a catastrophic impact on the outputs of an analysis: in the recent past this has led to numerous retractions or corrections,^1 2^ and it is likely that many coding errors go unnoticed. On some teams a single person may be responsible for writing all the code for a project. Code review typically involves a separate person examining the code, and sometimes running it, in order to spot issues. It aims to guard against error, and provide a useful opportunity for feedback or suggested amendments to improve the efficiency and readability of the code. Some research groups have implemented code reviews and have openly recommended this practice because of the benefits in quality and reproducibility.^10^ We believe code review is essential and hard work and reviewers should be acknowledged as full members of the study team.

##### How to do code review

There is no one method for code review; however in general it is best to review often, and not at the end of the project, and for both the researcher and the code reviewer to have clear expectations of what code review will entail. For example, does it include running the code entirely or simply looking and commenting on the code. Google has produced some guidance^11^ on how to think about and implement successful code review practices.

Some groups find it effective to use a ‘*buddy’* system where all code and outputs are checked by at least one other knowledgeable member of the study team for bugs and suggestions made for simple improvements. This can involve looking over a pull request, or checking an entire project to ensure it runs as expected. When you feel confident that your code does what is intended you can share it with the wider community which will ideally generate even more review and feedback. Code review is also one of the benefits of making code publicly available: having your code published enables other research teams as well as peer reviewers to assess the analytical code underlying any given study for accuracy. Even a cursory code review is better than none at all.

### Code sharing

Sharing the code that underlies your analyses is a quick, cheap, and easy way to provide transparency into your methods. Your code can usually be shared without many of the concerns around privacy and disclosure that can complicate data sharing. Other researchers working in the field can re-use and learn from code, with credit, for their own projects: this increases the efficiency of research, and may open the door to new collaborations. In open source software development it is standard practice for others to offer suggestions, improvements, or entirely new features to existing repositories. Making your code available may be the first steps towards future collaborations and making a more generalizable tool for the wider research community.

#### How to share code

Code can be shared in a variety of ways: the simplest option is to share code in an appendix to a paper; however it is better to use one of the free software development platforms, such as GitLab or GitHub, which provide additional benefits and usability to interested users as discussed above. These services allow users to develop and share code in a “repository”, which can be thought of as a project folder for each piece of work. In addition, users can interact with these platforms through simple graphical user interfaces, which is useful for those unfamiliar with working at the command line of an operating system. These platforms are indexed by major search engines meaning that your work is also more likely to be *found*. After uploading your code you can apply appropriate licenses that allow re-use of the software with or without restriction, modification, or citation. It is also easy to generate a digital object identifier (DOI) for specific versions of your code released through GitHub, by archiving through a service such as zenodo. GitHub also recently added support for citations files added directly to repositories.^12^ In our view researchers should always cite other researchers’ code when re-using it, or deriving insights from it: however as a formality we tend to use the MIT licence.^13^

#### Libraries

Useful functions, and their associated unit tests, often outgrow individual projects, and build a broader user-base. When they do, more experienced programmers move them into reusable code “libraries” and share them through package indexes or archive networks. By creating a library, researchers contribute to the broader research community. This more advanced variety of code sharing is common in many areas of scientific research, such as Geographic Information Science, but it is less common at present in health data research.^14–16^

##### How to create a library

Programming language communities have developed the tools to create and share code libraries easily through package indexes or archive networks. Python, for example, has PyPI, or the Python Package Index; R has CRAN, or the Comprehensive R Archive Network.

## Discussion

We hope this introduction into some of the basics of software development best practice is helpful to researchers of various levels of coding experience. Implementing the practices that fit your group’s workflow can increase productivity, facilitate open collaboration with the larger community, and ultimately lead to higher quality research. Importantly, in other disciplines, sharing code with good documentation has already been seen to produce quality and efficiency benefits for the wider research community.^17 18^

We recognise that there are barriers to embracing these practices: trying to do more with your code, beyond simply scripting out the analysis, can be intimidating; good code can be under-appreciated; and implementing these concepts in your own work may require familiarising yourself with new tools, jargon, and ways of thinking. A key area for development should be establishing communities of practice in research software to empower and educate researchers to use the tools that are available, in a way that works with their domain and team. Like-minded analysts with the UK NHS, for instance, have established an NHS R community to share knowledge, tools, and guidance among their peers.^19^ Software Carpentry and Data Carpentry have sought to do the same by running an introductory course followed on by support to run monthly engagement to develop a local community of practice[20%5D. Senior leadership buy-in to the value of these communities has been key to getting them running. Online forums such as StackOverflow have been set up by software developers to allow people to ask questions about how to solve problems when writing and implementing their code. These contain a knowledge-base of thousands of answered questions covering a wide array of topics and domains with the ability to ask new questions if yours isn’t covered.

Funders and journals may not fully appreciate that a well maintained and widely used open source library is as valuable as a high profile publication. We anticipate that research funders and leaders will increasingly recognise the value of software and its tools to the quality and efficiency of research.^21–23^ Journals could consider mandating code sharing at the time of publication and even simple moves such as establishing a software policy for the journal would encourage code to be shared.

## Conclusion

We strongly believe that researchers should aim to embrace modern best practice around software development because increasingly, in the era of data-driven research, research *is* software development. For this to occur, funders and journals need to buy-in to its value, and encourage individuals and teams to adopt the tools and techniques employed by the software development community.

## Data Availability

Data sharing not applicable as no datasets generated and/or analysed for this study.
